# A neuropsychological rehabilitation service delivery model for South African adults with acquired brain injury (RSDM-SA)

**DOI:** 10.3389/fresc.2023.1175963

**Published:** 2024-04-12

**Authors:** Noorjehan Joosub, Gert Kruger, Pieter Basson

**Affiliations:** Department of Psychology, University of Johannesburg, Johannesburg, South Africa

**Keywords:** acquired brain injury, rehabilitation, LMICs, accessibility, neuropsychology

## Abstract

In low- and- middle- income countries (LMICs) such as South Africa, a high number of Acquired Brain Injuries (ABIs) and a lack of accessibility to healthcare lead to many survivors of brain injury not receiving the level of healthcare and rehabilitation required. Further, in LMICs life-saving or acute care is prioritized with an inadequate focus on the lifelong effects of ABI. This study used Program Theory to develop a Rehabilitation Service Delivery Model for South African Adults with Acquired Brain Injury (RSDM-SA) that caters for the unique nuances of a resource-constrained and culturally diverse context. The RSDM-SA has four interdependent levels, namely (i) Integration of Relevant Aspects of Explanatory Frameworks (ii) South African Contextual Influences on the Model (iii) Systemic Role players Necessary for the Model and (iv) Evidence-Based Guidelines in a Holistic Rehabilitation Process. The Model is a valuable resource in guiding future research endeavors and its contribution lies in the Model's focus on quality, accessibility, relevance, and efficiency, all of which are needed in healthcare internationally.

## 1 Introduction

Survivors of acquired brain injury (ABI) often have an incomplete recovery when discharged from medical care, due to the chronic nature of the condition, and their families and caregivers struggle to cope with patients' residual impairments (1). Neuropsychological rehabilitation (NR) focuses on assisting ABI survivors who have cognitive, emotional, or behavioral deficits to live up to their potential in all facets of their everyday functioning (2). The most common deficits after ABI include cognitive impairments, behavioral changes, emotion dysregulation, and executive dysfunction, with an increased risk of premature mortality (3).

South Africa is classified as an upper middle-income country according to the World Bank, however, it also has one of the most unequal societies in the world (4). Rehabilitation after ABI is not offered as a standard procedure in South Africa due to resource constraints and skill shortages (5). The incidence of ABI in LMICs tends to be higher due to a lack of law enforcement, high levels of violent crime, and motor vehicle accidents (6). Further the emotional, cognitive, social, and vocational consequences of ABI tend to be more severe and debilitating in LMICs due to a lack of infrastructure, a lack of adequate acute and post-acute healthcare services, and the lack of support available for people with disabilities and their families. In contrast, intensive neuropsychological rehabilitation (NR) programs are available in developed countries (7). Studies on the incidence and impact of ABI in South Africa (8–12) identify the consequences of ABI as a national health concern.

Existing rehabilitation initiatives in LMICs tend to focus on physical and mobility impairments, without a concomitant focus on the pervasive cognitive and psychosocial difficulties after ABI (11). The physical and mobility impairments experienced after ABI are often less of a contributor to the disability and frustrations experienced by ABI survivors than other factors, such as a lack of available NR services, geographical access barriers, societal discrimination, and psychological adjustments needed (13). NR services have not been prioritized by healthcare funders even in developed countries due to their perceived lack of efficacy (7).This leads to the practice where healthcare budgets are spent on interventions that appear more effective, as well as on injuries that appear more amenable to treatment and cure than brain injuries. However, there is strong evidence that NR improves patients' and their families' lives after ABI (14–17).

Barriers to the provision of adequate NR services in South Africa include the lack of evidence-based research for some of the impairments experienced after ABI, as well as the negative perception that damage to the brain is permanent and impervious to treatment (5). Coupled with socioeconomic deprivation, this lack of rehabilitation services may lead to ABI survivors turning to crime or becoming dependent on their communities. In this way, additional strain is placed on underprivileged communities and the cycle of crime and violence is perpetuated. This is particularly relevant in South Africa where most patients with ABI come from extremely deprived socioeconomic circumstances, have no medical insurance, and have low levels of education (11). These contextual factors are important to consider when developing NR interventions for ABI in South Africa.

Internationally, there is increasing awareness of geographical, social, and cultural factors involved in illness, requiring a shift from the biomedical model to a psychosocial-environmental model for NR (7). This has implications for the understanding of ABI, as well as for approaches to treatment. The dominant models of NR are, however, neither culturally aware, nor are they suited for resource-constrained contexts, such as South Africa (18). Consequently, the need exists for the development of a model of NR after ABI that is suited for the South African context.

The cultural-historical approach to neuropsychology posits that the cerebral bases of neurological functions are intricately interwoven with cultural, social and historical influences (19). One of the most prolific theorists in this area, Alexander Luria, conceptualized a dynamic neuropsychology where brain-behaviour relationships are not simply in response to organic processes but also adapt to social conditions (20). Therefore, Luria revolutionized localization theories of brain-behaviour relationships to incorporate more functional integrated systems, with the patient at the centre of the analysis.

Globally, there have been significant improvements in NR services for ABIs in the past few decades (21). This improvement is not reflected in South Africa where socioeconomic disparities in addition to a range of other factors leads to NR service delivery not to be prioritized (22). The majority of ABI survivors in South Africa are also discharged to underprepared families and communities which are not ready to face, and assist with, the changes that occur after an ABI (11).

A recent estimate reported 89,000 new cases of TBI are sustained in South Africa every year. The majority of these are due to motor vehicle, bicycle, or pedestrian accidents (50%), 25% due to falls, 20% due to violence and five percent are uncategorized. South Africa's homicide rate is nine times the global rate, leading to the possibility that many TBIs are as a result of violence (6). An audit at a hospital in Cape Town reported that approximately 24% of the 10,046 trauma cases resulted from head injuries, and further, that 82% of assault-related head injuries were suffered by young black and colored men. These patients typically received approximately ten days of acute care before returning to a home setting characterized by unemployment, fragmented families, high levels of alcohol abuse, poverty, and a lack of infrastructure to provide vocational or psychosocial services (11). Due to this increasing need and yet scarcity of resources for NR in the developing world, there is a need for innovation and more effective service-delivery models. With disparities of service existing within South African societies among different socioeconomic groups there needs to be an increased focus on quality and accessibility in the design of NR interventions (8).

## 2 Method

This study used Program Theory to develop a Rehabilitation Service Delivery Model for South African Adults with Acquired Brain Injury (RSDM-SA) that caters for the unique characteristics of the resource-constrained and culturally diverse South African context. The use of Program Theory is relevant in the field of NR as NR practitioners are criticized for not being specific enough about the active ingredients for change in their treatments (23). Program Theory aims to identify the active ingredients for change, and facilitate a systematic and explicit delineation of the development of treatment models and practices that feed back into clinical practice (24). Due to the intricate nature of rehabilitation after ABI, NR interventions are, by nature, complex interventions (25).

Many NR programs are guided more by political, demographic, and economic factors than by patient characteristics or the intervention's proven efficacy. Treatment decisions need to be supported by evidence-based research to improve patient outcomes and maximize benefits from public spending (26). Systematic reviews collate and analyze research findings to provide practice standards (substantive evidence), practice guidelines (probable effectiveness), and practice options (possible evidence) for practitioners (15). Therefore, when developing a model for NR, the incorporation of practices that have evidenced successful outcomes, where possible, is an important endeavor. At the same time, it must be acknowledged that a large amount of EBR emanates from Euro-Western settings and such EBR cannot be generalized to all contexts (20). Further, the complex nature of NR interventions, especially concerning holistic models that typically involve multiple simultaneous interventions, with varying levels of intensity, make the development of evidence-based recommendations a complex endeavor (15).

The three-phase program theory research design, described by Van Hecke et al. (27) for developing complex interventions, was used for developing the NR Model for South African Adults with ABI. This specific research design was selected due to its thorough and systematic focus and its suitability for the research questions. The three phases of the Van Hecke et al. research design for developing complex interventions are as follows:

### 2.1 Phase one: collection of the building blocks needed for the intervention

The role of theory is crucial in intervention design (28) as theory-driven interventions are based on an idiographic worldview that accommodates inter-individual differences (29). The sequential procedure described by Hidecker et al. (30) was used to incorporate guidelines from the vast body of literature for NR into the model. The beginning phase of the research design incorporated a thorough literature review that aimed to investigate existing literature about the focus of the intervention and available treatment modalities and was collated between January 2016 and June 2019.

### 2.2 Phase two: intervention design based on all the relevant information collected in phase one

Based on the information collated in Phase One, an initial service delivery model was constructed. The following interrelated guidelines for a theory-based intervention were adapted by Sidani and Braden (31) from Lipsey's (32) original version and were used in the intervention design: (1) Problem Definition, (2) Critical Inputs, (3) Mediating Processes, (4) Expected Outcomes, (5) Extraneous Factors, and (6) Implementation Issues. The first four guidelines focus on descriptive components and the last two on prescriptive components.

### 2.3 Phase three: validation of the intervention by expert evaluation

Two experts were approached to evaluate the initial model based on their years of expertise, recommendations from other practitioners, and their experience in NR. Criteria for selecting the experts included: specialized training in the field of neuropsychology, a minimum of ten years of post-qualification experience, and specific experience in applying their knowledge in the field of NR in South Africa. Of the proposed evaluators, Evaluator 1 had 24 years of experience in both South Africa and the United Kingdom. Evaluator 2 had 33 years of experience in the South African context.

Based on the above procedure a model was developed that has four interdependent levels and incorporated resource-relevance with evidence-based research.

## 3 Results

**Figure 1:**
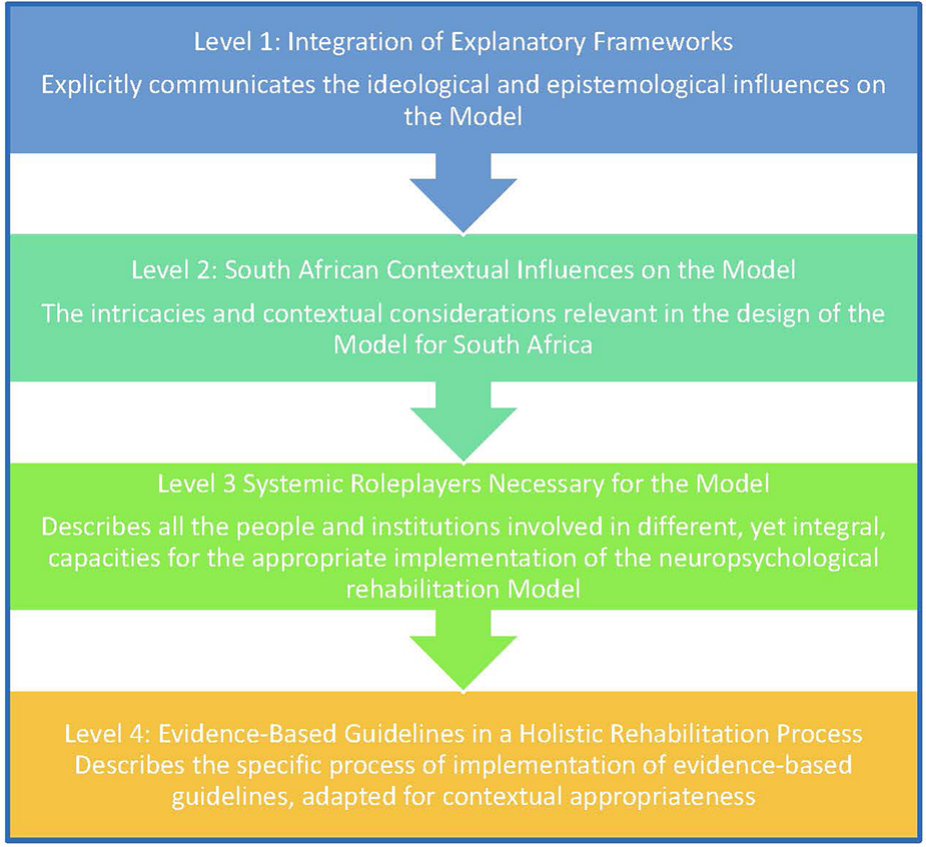
The four levels of the South African model of neuropsychological rehabilitation for adults after acquired brain injury.

**Figure 2:**
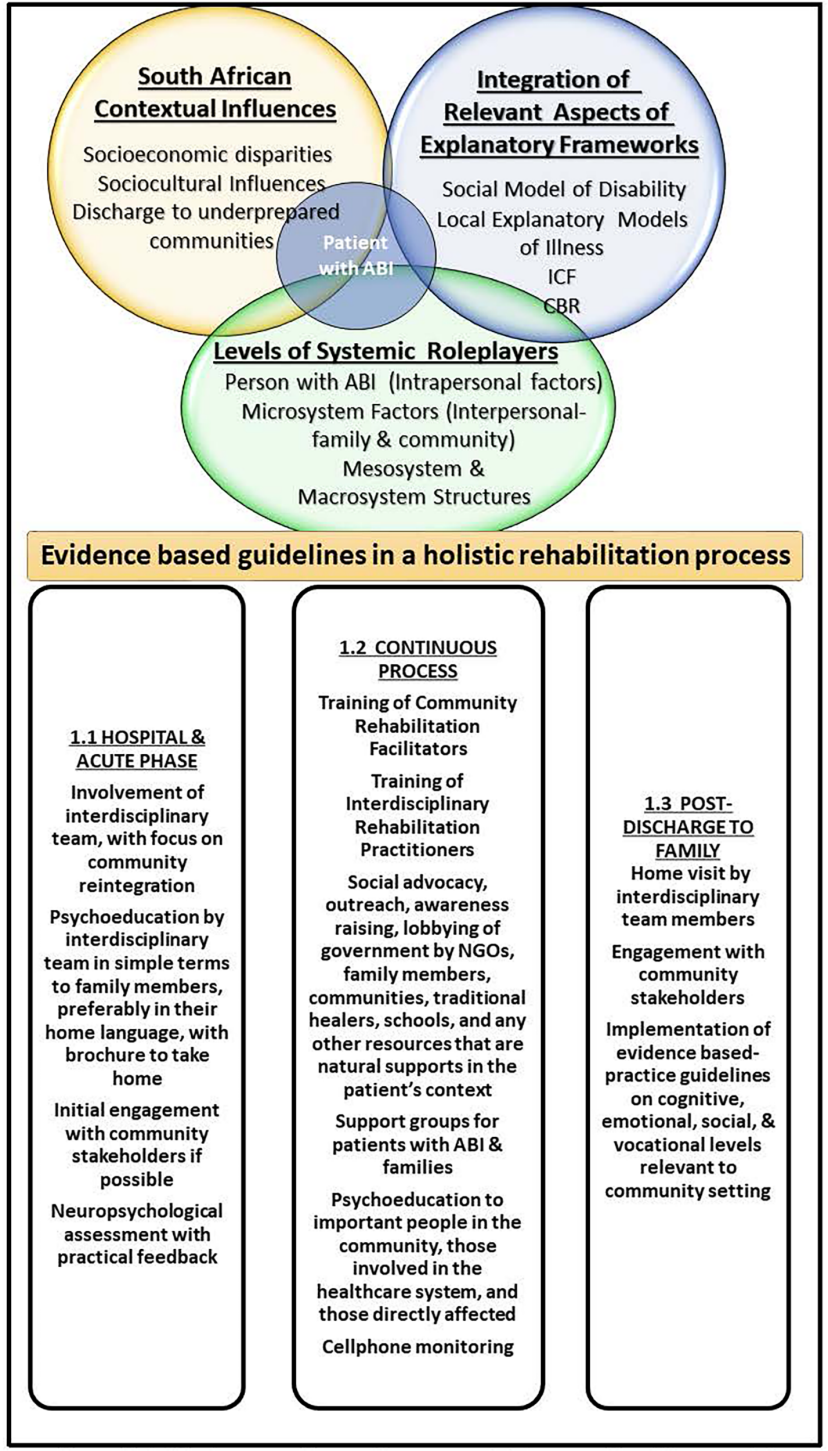
The rehabilitation service delivery model for South African adults with acquired brain injury (RSDM-SA).

Figure 1 below describes the different levels of the model, where Levels 1–3 explicate the relevant factors intended to inform the Model as a whole. Level 4 presents the NR process itself as a practical outcome from the factors and considerations presented in Levels 1–3. Figure 2 then goes into the details of what was included in each of the levels.

### 3.1 Level 1: integration of explanatory frameworks

**Figure 3:**
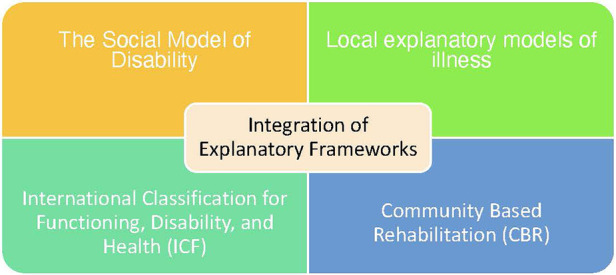
Explanatory frameworks that contributed to the model.

As depicted below in Figure 3, the explanatory frameworks that contributed to the Model were the Social Model of Disability (33), Community Based Rehabilitation (CBR) (34), local explanatory models of illness (35) and the International Classification for Functioning, Disability, and Health (ICF) (36). Each of these will be discussed below.

#### 3.1.1 Contribution of the social model of disability to the model

The premise of the Medical Model of Disability locates both the etiology and treatment of any functional impairments within the individual, whereas the Social Model of Disability looks to environmental antecedents and potential for treatment within the social environment (37). Within the Social Model of Disability, political activism and advocacy are integral to rehabilitation processes. In South Africa psychologists work in the assessment and diagnosis of ABI, but often do not get involved in rehabilitation due to a lack of funding, and may feel disempowered in their professional role (38). Language is a further discriminatory factor where the patient with ABI is labelled and the perceptions of ABI and its consequences are socially constructed. The vocabulary used in the dominant Western Medical Model, originating from North America and Europe, may be foreign and exclusivist to those from different cultures (39). Disability roles for ABI survivors in South Africa may lead to further disenfranchisement. For example, patients with aphasia often experience being spoken over or referred to in the third person, increasing their invisibility and lack of voice (40). These structural inequalities and discourses are present within South African NR settings as well, where professionals may lack a reflexive awareness of the power differentials present between them and patients. However, some limitations of the Social Model of Disability include that it does not account for differences in experiences of disability or intersectionality, and that it focuses extensively on changing society to the detriment of understanding the person with ABI's lived and embodied experiences of disability (41). Next the WHO's International Classification for Functioning, Disability, and Health (ICF) will be discussed regarding how it has guided the model.

#### 3.1.2 Contribution of the WHO's ICF to the model

Instead of conceptualizing disability in terms of the etiology of the impairment, the International Classification for Functioning, Disability, and Health (ICF) focuses on an ABI's impact on the patient's environmental participation, activities, and body structures and functions (42). This is relevant when working with South African patients with ABI as it allows practitioners to be aware of factors, such as premorbid personality characteristics, which are often neglected in linear, biomedical models (42). Regarding the ICF in relation to NR, in 2013, TBI ICF Core Sets for the rehabilitation of TBI were developed to assist understandings of impairments after TBI as well as to aid treatment planning (43). Important ICF indicators after ABI include social inclusion, productive activities, social integration, home integration, cognitive abilities, physical abilities, quality of life, behavioral functions, and emotional regulation (44). This is relevant to the South African context because of the prevalent misconceptions around ABI which hinder workplaces and communities from adequately accommodating patients with ABI. Family members, particularly in collectivist societies like those prevalent in some parts of South Africa, have a great willingness to assist, however this often results in overprotection which could impede the patient's functional recovery. In a qualitative study of work adaptation after ABI in South Africa, one participant with ABI reported that family members doing everything for him made him feel childlike (45).

One of the challenges encountered in using the ICF includes the complicated nature of the nested structure that entails some familiarity before one can use it efficiently. Further, there is potential for the ICF to be misused by professionals due to the amount of knowledge required and utilized, thereby decreasing the patient's privacy and autonomy (43).

#### 3.1.3 Contribution of CBR to NR after ABI in South Africa

The intricate relationship between poverty and disability is acknowledged in CBR, and therefore rehabilitation efforts should be cognizant of socioeconomic needs and resource constraints. This is useful in the South African context due to the high levels of poverty many patients with ABI face (6). Ideally, CBR envisions a partnership among all levels of the systems in society, such as government departments, academic teaching departments, NGOs, religious bodies and individual community members (46). This approach is relevant for the South African context as systemic change within the healthcare system cannot be implemented without a shared vision and collaboration among all the stakeholders involved, from macroeconomic policymakers to the patient with ABI themselves (47). Community Rehabilitation Facilitators (CRFs), usually those in the community who do not have employment or those with disabilities, are needed to form links between mainstream healthcare services and outlying communities, taking knowledge, skills, and empathy back to communities. Insiders in the community are more aware of, and respectful towards, the cultural and religious sensitivities of patients and their family members (48). In an in-depth qualitative study on the impact of CRF interventions from the perspectives of people with disabilities (PWDs) and their families, it was found that that the psychosocial components of CBR are just as valuable as the physical components (49). Home-based NR treatments have demonstrated cost-effectiveness without compromising clinical effectiveness (50, 51). The limitations of CBR include is that it requires governments to take executive control of the implementation of this strategy (46). A qualitative study of six CRFs in South Africa found that these workers encountered challenges such as a lack of training, poor communication among governmental departments, funding shortages, and a lack of resources (52).

#### 3.1.4 Contribution of local explanatory models of illness to the model

The Local Explanatory Models of Illness framework postulates that clinical knowledge needs to be combined with an understanding of local discourses and illness representations (53). Indigenous healing is an oft-neglected resource and valued tradition in African societies that has been marginalized by the hegemonic dominance of mainstream Euro-western medicine (47). The decolonization of NR needs to accommodate and promote local forms of knowledge (54). For example, in rural Shangaan communities in South Africa, symptoms of stroke are often the same as those of a type of bewitchment called *xifulana* (55). Surya (56) therefore asserts that a collaboration between cultural and societal strengths, and scientific NR insights, is the way forward for NR in the developing world. In African traditions music has spiritual, physical, and mental benefits (57, 58). This is particularly relevant for the multicultural situation in South Africa. For example, music is often integral to African healing ceremonies and religious traditions (57, 58), and there is EBR on the usefulness of music in NR interventions for difficulties with gait, verbal memory, and focused attention (59, 60).

South African explanatory models of illness allow for an understanding of contextual epistemologies that inform and enhance understandings of illness. However, difficulties may be encountered when attempting unified understandings of illness and further this framework runs the risk of reifying differences within cultures. Further, culture is not a static entity and therefore changes in cultural conceptions across generations of the same community are common. This may lead healthcare practitioners to erroneously ascribe homogenous illness representations to different cultural groupings within the same community.

The explanatory frameworks discussed above provided a metatheoretical foundation that guided the Model. The following section will discuss the specific contextual influences that also had a major influence on the development of the Model.

### 3.2 Level 2: South African contextual influences on the model

The second level of the proposed Model focuses on contextual realities influencing NR in the South African context. These include socioeconomic disparities, sociocultural influences, and the discharge of patients to their underprepared communities.

#### 3.2.1 Socioeconomic disparities

The oppressive political history of apartheid left an enduring legacy of fragmented healthcare systems and skewed geopolitical priorities. South Africa has high levels of unemployment, poverty, crime, and violence, a lack of infrastructure, fragmented families (6, 11) and high levels of socioeconomic inequality (5). High levels of illiteracy also impact NR processes (4). A lack of availability of cross-culturally appropriate test materials, many practitioners not being proficient in indigenous languages, neuropsychological assessment settings not being conducive, and a lack of expertise that could lead to the development of culture-fair tests also hinder the process (4). Solutions for the challenges mentioned above include the use of patient and family reports, direct observations, and the use of dynamic assessments that are more culture-fair, and can be used to better understand the patient's profile of strengths and weaknesses (61). The majority of ABI patients in South Africa are male, from very low socioeconomic circumstances, with no access to medical insurance, and low levels of education (55). Alcohol abuse has been correlated with risk for sustaining a TBI in South Africa. There is a lack of accessibility of NR services available to the majority of the population due to fragmented healthcare systems (62). It is necessary in LMICs, like South Africa, for professionals and internship psychologists in major urban areas to volunteer their services for a time in outlying areas and to train support workers in these areas to improve the accessibility of mental health services (47). Group interventions and outreach initiatives could be invaluable in increasing accessibility for NR. There needs to be collaboration among primary healthcare institutions and tertiary institutions, as well as a prevailing discourse within healthcare, to serve those who are underprivileged and lack adequate services. Psychologists should be involved in dismantling stigmatized discourses through outreach health promotion, advocacy, policy, and illness prevention services that allow those in need to identify when they need help and feel empowered to ask for such help (47).

#### 3.2.2 Sociocultural influences

The understanding of cultural influences on perceptions of etiology and rehabilitation-seeking behaviors is an important resource in assisting NR interventions (63, 64). Human social life has been recognized as far back as Luria's social-historical neuropsychology as integral to understandings of brain-behaviour relationships (20) In African cosmology, misfortunes and disasters do not happen by chance; they are linked to mystical powers, witchcraft, or ancestral spirits. The sociocultural meaning behind the ABI is significant and could lead to attempts to appease the ancestors or consult a traditional healer who could consult with the ancestors. Some family members may expect the person with ABI to heal from the injury instead of seeing it as a lifelong reality (65). In many collectivist cultures, such as those predominant in South Africa, the treatment of physical ailments is related to the restoration of spiritual balance (17). Religion serves as a coping mechanism for caregivers who believe that the ABI is God's test, and support can be gained from their spiritual institution and its congregants (65).

#### 3.2.3 Discharge of patients to underprepared communities

Due to the lack of quality post-injury healthcare accessible to the majority of the South African population (55), many ABI survivors are discharged to family and community members after being medically stabilized (11). These family members often have not been exposed to any psychoeducation around ABI and, in the short-term, are just relieved that their relative is still alive. It is in the post-acute phase however, that family members realize that there are enduring impairments after an ABI that still need management (11). The focal point of NR therefore moves from inpatient institutions to communities in South Africa, and family members, community members, and support workers may have to act as co-therapists (40, 66). Many older community members, in the spirit of Ubuntu, are open to caring for patients after ABI (62), however there is an increasing culture of individualism in younger generations (67, 68) which may lead to less support available to patients in their communities. The specific home setting of the patient thus needs to be taken into account in NR planning, utilizing natural supports and cultural strengths (56).

The next section discusses Level 3 of the proposed model. Here the focus is on those people and institutions integral to the successful implementation of the Model which have been guided both by the explanatory frameworks and the South African contextual influences discussed above.

### 3.3 Level 3: systemic role players necessary for the model

Many different levels of collaboration are needed to successfully implement the proposed NR Model. In its ideal form, CBR envisions a partnership among all levels of the systems in society, from government departments, academic teaching departments, NGOs, and religious bodies, to individual community members (69). A focus on transferring ownership and responsibility of the NR process to local stakeholders, who will be most instrumental in ensuring the success of the NR Model, is needed (70). In this section, intrapersonal factors relevant to the patient with ABI, the microsystem and role replacement, as well as important mesosystem and macrosystem structures are discussed in relation to their relevance for the Model.

#### 3.3.1 Intrapersonal factors relevant to the patient

Many theorists indicate how crucial patient motivation and engagement are for the success of NR interventions (60, 71–74). Cognitive rehabilitation interventions need to take into account the importance of patient motivation, the patient's subjective experiences and beliefs that influence prognosis, as well as the patient's phenomenological experiences of rehabilitation (75). Patients have to be active participants within the rehabilitation process (40). This involves working with patients' levels of motivation, their appraisals of their self-efficacy, their ability to sustain effort, and their levels of self-awareness and self-reflection (71). The person with ABI has been placed at the center of the RSDM-SA to emphasize the importance of taking into account their unique needs.

#### 3.3.2 The microsystem and role replacement

Due to the lack of post-acute services available for most patients, once they are medically stable, they are discharged to underprepared families or their communities (62). Home-based learning within the context of the patient's everyday life allows for rehabilitation tasks to be generalized to daily activities, and helps patients and families to be prepared to deal with future problems (61). There is a need for NR to start from the acute care setting, to focus on early intervention and prevention of difficulties before the patient returns home, and to be a lifelong process (76). Cognitive screening of patients before discharge would assist in identifying rehabilitation focus areas and facilitate patient-specific training for caregivers and families (77). Family members also need ongoing support to assist them with difficulties that may arise in the rehabilitation process (40, 62). Community service psychologists can be involved in facilitating these processes (47).

#### 3.3.3 Important mesosystem and macrosystem structures

In the Circles of Support framework, networks of support include friends, neighbors, family members, volunteers, community services, paid caregivers, and professionals (78). For the successful implementation of the NR service delivery Model, all of these stakeholders should have a shared vision of collaboration and local ownership, as well as an understanding of the complexities of healthcare systems on the ground (79). CRFs are valuable resources and can take knowledge, skills, and empathy back to communities, and can train interdisciplinary teams on local knowledge (60). Links with the educational, employment, and labor sector are also vital to ensure, where possible, a return to productivity after an injury (36). Community service psychologists could be valuable in in training CRFs in outlying areas and empowering them to assist others such as family members and caregivers of patients with ABI (47). NGOs, policy and governmental regulations, and informal community mechanisms and traditions are further levels of this intricate system of stakeholders that could assist the NR process (70). Psychologists involved in NR services could collaborate with important community leaders such as respected elders, religious clerics, and traditional healers to improve accessibility of support and services (55). Places of worship, NGOs, and social welfare agencies are examples of existing infrastructure that can be used as bases for support groups and community rehabilitation efforts (69). National frameworks need to emphasize the need for training in ABI across all practitioners such as psychologists, social workers, physiotherapists, and occupational therapists.

The following section details the guidelines for implementation of the RSDM-SA. These guidelines incorporate practice guidelines and recommendations from evidence-based research and have been adapted for the South African context.

### 3.4 Level 4: evidence-based guidelines in a holistic rehabilitation process

Evidence-based guidelines were collected from the literature based on the sequential procedure described by Hidecker et al. (30). These evidence-based guidelines are divided into guidelines for cognitive, psychological and vocational rehabilitation as well as for the holistic rehabilitation process.

#### 3.4.1 Implementation guidelines of evidence-based interventions in the South African context for cognitive rehabilitation

Compensatory strategies are generally accepted to be more effective than remedial or retraining approaches in cognitive rehabilitation (80). Families and CRFs could be assisted to help patients brainstorm compensatory strategies like writing things down to assist memory or doing a cognitively demanding task for short periods to assist attention. Cognitive retraining should be domain-specific, working with one cognitive domain at a time (81). Rehabilitation professionals and CRFs could also be taught to work with one functionality, such as short-term memory or attention, at a time for the best results. Such specific interventions need to be tailored to the age of the ABI survivors, their levels of education, and the extent of their injuries (82).

Direct attention retraining in conjunction with teaching metacognitive strategies has been found to be effective for improving compensatory strategies for attention impairments (15). For implementation in the South African context, patients should be taught to be metacognitively aware of attention processes before attention training commences. Attention training can incorporate simple exercises such as reading a book for 15 min at a time. Metacognitive strategies applied to reading a book would be to become aware of when you are not focusing on what you are reading any more, and then to take a break.

Computerized programs can also be helpful for attention as long as they are administered under direct supervision (15). In most South African contexts, access to computers and specialized rehabilitation software is a privilege, therefore these will have to be substituted with easily accessible cellphone apps, such as taking photos of your day to cue autobiographical memory, or ecological home-based tasks such as cooking a meal.

Unilateral neglect has been shown to be assisted by limb activation devices (83), and visual scanning training (75). These are practical approaches that occupational therapists can share with CRFs to assist with unilateral neglect after stroke, for example by encouraging the patient to brush their teeth with the hand that has been affected by the stroke.

Difficulties with gait have been found to be assisted by music interventions (59). Neurologic Music Therapy assists verbal memory and focused attention (17). These guidelines should be incorporated with existing musical practices for traditional African healing and religious rituals, such as walking to the rhythm of drumming for example (57, 58).

Language training assists aphasia after stroke (81) and therefore speech therapists in community contexts can train caregivers and CRFs to do simple language exercises in the home language of the patient. Cognitive-linguistic therapies, pragmatic conversational skills, group-based interventions, and computer-based interventions with clinician-guidance, help both language and communication deficits after ABI (15). These groups and skills can be initiated by professionals and interdisciplinary teams who then train caregivers and CRFs to facilitate them in a sustainable manner, such as CRFs facilitating groups to improve social communication skills. Linguistic abilities vary based on acculturation and local dialects need to be prioritized (82).

There is strong evidence for the effectiveness of external memory aids that enhance functionality without necessarily improving underlying memory abilities (84). These external memory aids need to be directly linked to ADLs (66). During the psychoeducation phase, professionals can encourage caregivers and CRFs to teach patients to rely on aids such as cellphones and notebooks to remember to undertake personal hygiene, medication-related, and food preparation activities. Internal memory strategies assist patients with mild memory impairment (15). Simple techniques like mnemonics to assist memory can be taught to caregivers and CRFs to help them assist patients.

A combination of external aids, such as cellphones and calendars, with internal strategies such as self-monitoring, have been found to allow for the most improvement in prospective memory functioning (80). Caregivers and CRFs need to explain to patients the impact of their memory difficulties and encourage that aids are utilized in conjunction with a metacognitive awareness of memory difficulties. For example, patients could try to write the name of a new person when meeting them and test themselves to see if they remember the name half an hour later.

SenseCam (85) and NeuroPage (86, 87) have demonstrated effectiveness in assisting memory difficulties in Western contexts but are expensive technological aids not suitable for resource-constrained contexts like South Africa. However, if similar applications are developed at minimal cost, they would be more accessible Cost-effective strategies, such as taking photos of one's day to remember it and setting alarms as reminders on cellphones, can also be used as compensatory strategies for memory impairment.

Cappa et al. (87) found evidence for the probable effectiveness of errorless learning techniques, process-oriented training, self-instructional recall techniques, and spaced recall techniques, to assist memory functions after ABI. During the psychoeducation phase, professionals can empower caregivers and CRFs with these strategies to assist patients with memory difficulties. For example, CRFs can monitor patients when learning new skills to ensure errorless learning.

Metacognitive strategies, including self-monitoring and self-regulation exercises, were found by Cicerone et al. (75) to assist with emotional self-regulation, problem-solving, planning, organizational difficulties, and deficits in memory, attention, and neglect after TBI. Caregivers and CRFs need to work with the patient's level of insight to explain to them where possible the importance of self-regulation. An example of such a metacognitive strategy is keeping a journal to monitor one's mood.

SMS technology has been found to be useful in the prompting of rehabilitation goals (88). SMS technology is inexpensive and accessible, and can be used by professionals, caregivers, and CRFs, to assist and monitor patients. For example, SMS reminders can assist patients adhere to medication regimens.

Problem-solving Therapy has been associated with improvements on tests of problem-solving and general intelligence when applied in a group setting (89); and studies have demonstrated the effectiveness of problem-solving therapy for executive function deficits (60). During the psychoeducation phase, professionals can empower caregivers and CRFs with these strategies to assist patients with executive function difficulties. For example, a CRF can assist an ABI patient with a list of pros and cons when making a difficult decision. There is strong evidence for the use of methylphenidate to improve processing speed after ABI (90). Caregivers and CRFs should look out for difficulties with attention and response speed and refer to a psychiatrist where necessary.

#### 3.4.2 Implementation guidelines of evidence-based interventions in the South African context for psychological rehabilitation

CBT and seretonergic antidepressants appear to be effective for psychological difficulties after ABI (91). Professionals can impart simple CBT exercises to caregivers and CRFs to assist patients, for example patients could keep a positive behavior record. Patients' moods should also be monitored for potential referral to a psychiatrist for antidepressants. Physical exercise has been found to improve mood (7) and patients should be encouraged to exercise where possible and group exercise interventions can be used for motivation.

Disinhibited or aggressive behavior should be addressed to decrease the likelihood of criminal activity (92). Caregivers and CRFs could be taught behavior management strategies for such behaviors that would deter their escalation such as walking away from stressful situations and other emotion regulation strategies. Focused, intensive and effective psychotherapy modalities are more useful than long-term, costly interventions such as psychodynamic therapy (13). CBT is a short term and focused therapy that can be reinforced by CRFs and caregivers.

#### 3.4.3 Implementation guidelines of evidence-based interventions in the South African context for social rehabilitation

Group therapy has been found to have robust evidence for its effectiveness to assist with language impairments and executive functioning difficulties, and to improve memory and social communication skills after ABI (93). Where possible, group interventions can be initiated by professionals to be facilitated by CRFs, caregivers, or those with an ABI who can do so. Where possible, collaborations with NGOs like Headway would also assist these processes.

In collectivist cultures, support groups for those affected and their families can be a valuable resource that strengthens individual coping skills and collective resources after an ABI (61). Support groups are cost-effective in under-resourced settings (47) and have been found to improve patient and caregiver wellbeing (94). Where possible group interventions can be initiated by professionals to be facilitated by CRFs, caregivers, or those with an ABI who can do so. For example, patients with ABI could meet once a week to discuss and share strategies for dealing with memory difficulties.

Evidence exists for the beneficial effects of community rehabilitation that is interdisciplinary, that supports the family and caregivers of the patient, and that is implemented even many years after the injury (66). Patients, family members and caregivers should be supported through group therapy, support groups, and professional assistance as far as possible. For example, caregivers should be encouraged to form support groups that could meet monthly to assist one another.

#### 3.4.4 Implementation guidelines of evidence-based interventions in the South African context for vocational rehabilitation

The following factors predict successful return to work: access to vocational services, improved global cognitive functioning, pre-injury occupational status, intact executive functioning, and perceptual abilities (95). Where possible, career counsellors, Counselling Psychologists or Industrial Psychologists should train caregivers and CRFs to assist the ABI patient to establish realistic feasibility of finding employment or getting involved in meaningful activities.

The following section describes the specific process of implementation of evidence-based guidelines, adapted for contextual appropriateness.

#### 3.4.5 Implementation guidelines of evidence-based interventions in the South African context for the holistic rehabilitation process

Systematic reviews indicate strong evidence for the beneficial effects of early provision of psychoeducation after ABI (96). Therefore, important stakeholders should receive psychoeducation, such as primary and secondary education learners, teachers, parents, and workplaces or employees, those involved in the healthcare system such as GP's, clinics and nursing staff, social services, and hospitals, and those directly affected such as clients, families, and those who have not received acute care before.

NR interventions should begin from the time of the first admission of the patient into the hospital (26). This is where the family members or significant caregivers should receive psychoeducation on the different trajectories of recovery after ABI, and how to assist the injured. Psychoeducation should ideally be in the language that the patient is familiar with and should include a written pamphlet or book detailing important information about ABI, and the prognosis for recovery that can be taken home for later reference. Interdisciplinary team members need to undergo constant training for the updating of their knowledge and skilling in the latest evidence-based practice guidelines.

It is recommended that before discharge from hospital patients undergo a cognitive screening to assess for relevant areas for cognitive rehabilitation and caregiver support (77). The results of the screening should include detailed guidelines on specific areas for improvement that could assist caregivers of the patient to take ownership of the NR process. Ideally, the results of this assessment should be discussed with the patient and their family before discharge with specific pointers on how to assist the patient with adjustment to their home environment.

Once home, a home visit by a healthcare professional skilled in NR is necessary to determine the natural supports and strengths in the environment that could facilitate the NR process (69). This visit would include a discussion with all individuals involved with the patient, and referral to local support groups for both the patient and their family members should be encouraged (79). All members of the family or community who are affected should be assisted during this adjustment phase. Any other additional support,such as healthcare professionals doing community service in the area or lay volunteers and CRFs, should also be pointed out to the patient and their family at this stage of the process.

Community members, religious leaders, traditional healers, CRFs, neighbors, and any other important stakeholders should be engaged during this home visit to assist with the patient receiving the optimal amounts of support and understanding needed (62). Evidence-based guidelines should be shared in simple terms containing practical guidelines with these natural support structures, based on the difficulties the patient is facing. An example would be the use of external aids for cueing memory. During this meeting, the focus should constantly be on improving the patient's levels of participation, activity, and functioning within their context or society.

Follow-ups by healthcare professionals such as neuropsychologists, occupational therapists, and speech therapists should consistently occur every six months assisting those involved with the ABI patient to strategize, improve foci for possible vocational rehabilitation, and look for areas of improvement on the cognitive, social, emotional, or vocational levels. Cellphone technology, such as SMS, can be used to monitor progress in between follow up visits (88). Support workers and CRFs should be constantly involved in advocacy and awareness-raising, particularly with regards to social inclusion and neurodiversity in the workplace.

## 4 Discussion

The emphasis on creative and resourceful NR interventions could benefit those with ABI and their families in both developing and developed countries, as healthcare funding is being reduced globally. The RSDM-SA Model prioritizes quality, accessibility, relevance, and efficiency, all of which are needed in healthcare across the world. A further contribution is increasing the body of knowledge on evidence-based practices in NR, with the systematic process followed in collating the guidelines beneficial for those looking to develop evidence-based guidelines in different contexts.

However, there are also limitations of the RSDM-SA. One of the limitations of the explanatory frameworks is that they are based implicitly on the assumption that societies are essentially humanitarian and altruistic, which is perhaps their biggest flaw. When survival is a priority, assisting those who are more vulnerable in a community may not be prioritized. There are multiple levels of societal disadvantage within South Africa, most emanating from structural inequality (97). For example, one of the explanatory frameworks, the Social Model of Disability, emanated from the U.K., which, compared to South Africa, is a WEIRD (Western, Educated, Industrialized, Rich, and Democratic) context. Therefore, while the Social Model of Disability assumes that those with difficulties such as ABI are oppressed by those in a position of privilege, in South Africa there are very few people within society who are in a position of privilege (98). South Africa, unlike the U.K., has a lack of access to basic resources and infrastructure such as food security and healthcare accessibility for most of the population. However, if there are interventions to increase accessibility to resources for the whole community, then the model will have managed to achieve beneficial effects for the person with ABI, as well as their community (99).

A further limitation is that by emphasizing cultural contributions, the RSDM-SA assumes homogenous conceptions of culture. Local explanatory models of illness are constantly in flux and need to be flexible enough to accommodate diverse and evolving contexts (100). A limitation of community-based rehabilitation as an explanatory framework is that it requires the South African government to take executive control of the implementation of this strategy (46). There is a lack of structure and emphasis on the allocation of necessary financial resources in the CBR strategy produced by the South African government (46).

Regarding South African contextual influences, the past 24 years of democracy in South Africa have been a time of flux, with many positive and negative developments impacting on healthcare (47). One of the limitations of acknowledging that patients are often discharged to underprepared communities is that it does not challenge the reality that some patients do need more specialized care, and that many families are so involved in struggling to survive that they do not have the time or economic resources to devote to caregiving. Further, healthcare professionals in the public sector are often overworked so they may not have the time for the devolution of specialist skills (101).

Regarding the Systemic Role players Necessary for the Model, role replacement is not simple as many families and communities are so involved with survival that they cannot cope with the burden of rehabilitation tasks. Healthcare professionals in the public sector are similarly overworked and therefore may not be able to support role replacement when needed (101). The involvement of extended structures is necessary to counterbalance the individualism of the biomedical model, however this may be difficult to implement in practice (79).

Lastly on the level of Evidence Based Guidelines in a Holistic Rehabilitation Process, on a macrosystem and mesosystem level, the stressors on healthcare practitioners are also great and will affect their ability to train caregivers within the patient's context. The availability of CRFs is a strength however, they may be overworked. There are also language barriers that will need to be overcome for cognitive rehabilitation techniques to be communicated. There is once again a need for macrosystem support to overcome these barriers. Further, evidence-based guidelines will constantly need to be updated as new research is undertaken.

## 5 Conclusion

As societies across the world become more integrated, the recognition of psychosocial, spiritual, cultural, and environmental influences on healthcare will become more prominent. With increased integration, mobility, and exchange across societies internationally, healthcare interventions are increasingly being recognized as being complex to implement. Focusing on quality, accessibility, efficiency, and relevance in healthcare interventions necessitates that interventions consider multiple factors and influences that are necessary for their success. Rehabilitation after ABI entails a focus on the patient's lifelong quality of life and flexibility in response to dynamic and evolving life challenges. Each presentation of ABI is unique and therefore designing complex interventions to deal with these challenges is necessary. The RSDM-SA Model serves as a foundation for future research to develop and improve. It is envisioned that future research could focus more closely on the implementation of the RSDM-SA Model in different settings in South Africa and, if possible, in other developing countries. Both qualitative and quantitative approaches would be useful in exploring the implementation of the RSDM-SA Model.

## Data Availability

The raw data supporting the conclusions of this article will be made available by the authors, without undue reservation.
